# Chemical Characterization, Antilipidemic Effect and Anti-Obesity Activity of *Ludwigia octovalvis* in a Murine Model of Metabolic Syndrome

**DOI:** 10.3390/plants12132578

**Published:** 2023-07-07

**Authors:** Dulce Lourdes Morales-Ferra, Miguel Ángel Zavala-Sánchez, Enrique Jiménez-Ferrer, Celeste Trejo-Moreno, Manasés González-Cortazar, Claudia I. Gamboa-Gómez, Fernando Guerrero-Romero, Alejandro Zamilpa

**Affiliations:** 1 Centro de Investigación Biomédica del Sur, Instituto Mexicano del Seguro Social, Xochitepec 62790, Mexico; lou_ferra@hotmail.com (D.L.M.-F.); enriqueferrer_mx@yahoo.com (E.J.-F.); gmanases@hotmail.com (M.G.-C.); 2 Doctorado en Ciencias Biológicas y de la Salud, División de Ciencias Biológicas y de la Salud, Universidad Autónoma Metropolitana (UAM), Mexico City 04960, Mexico; 3 Departamento de Sistemas Biológicos, División de Ciencias Biológicas y de la Salud, Universidad Autónoma Metropolitana (UAM), Mexico City 04960, Mexico; 4 Facultad de Medicina, Universidad Autónoma del Estado de Morelos, Cuernavaca 62350, Mexico; ctm@uaem.mx; 5 Unidad de Investigación Biomédica, Instituto Mexicano del Seguro Social, Canoas 100, Durango 34067, Mexico

**Keywords:** metabolic syndrome, hypercaloric diet, FFA, *Ludwigia*, corilagin, ellagic acid, gallic acid

## Abstract

*Ludwigia octovalvis* (Jacq.) P.H. Raven is widely used in traditional medicine for different illnesses, including diabetes and hypertension. However, its impact on lipotoxicity and metabolic syndrome in vivo has not been addressed. Therefore, the aim of this study was to evaluate the effects of this plant on the metabolic syndrome parameters in a C57BL6J mouse hypercaloric diet model. *L. octovalvis* hydroalcoholic extract and its ethyl acetate fraction (25 mg/kg/day) were used for sub-chronic assessment (10 weeks). Additionally, four subfractions (25 mg/kg) were evaluated in the postprandial triglyceridemia test in healthy C57BL6J mice. The hydroalcoholic extract and ethyl acetate fraction significantly decreased body weight gain (−6.9 g and −1.5 g), fasting glycemia (−46.1 and −31.2 mg/dL), systolic (−26.0 and −22.5 mmHg) and diastolic (−8.1 and 16.2 mmHg) blood pressure, free fatty acid concentration (−13.8 and −8.0 μg/mL) and insulin-resistance (measured by TyG index, −0.207 and −0.18), compared to the negative control. A postprandial triglyceridemia test showed that the effects in the sub-chronic model are due, at least in part, to improvement in this parameter. *L. octovalvis* treatments, particularly the hydroalcoholic extract, improve MS alterations and decrease free fatty acid concentration. These effects are possibly due to high contents of corilagin and ellagic acid.

## 1. Introduction

Metabolic syndrome (MS) is a cluster of interconnected physiological alterations which includes insulin resistance (IR), obesity, a pro-inflammatory state, atherogenic dyslipidaemia, arterial hypertension (HT), and a prothrombotic state [1]. Together, these alterations increase the risk of developing chronic-degenerative diseases such as diabetes mellitus [2].

The most widely accepted of the hypothetical mechanisms for the underlying pathophysiology of MS is IR with fatty acid hyperflux [3]. Chronic exposure to supraphysiological glucose levels, which is commonly found in IR states, can lead to glucotoxicity [4]. Meanwhile, hyperflux of free fatty acids (FFA) contributes to the phenomenon of lipotoxicity and the accumulation of ectopic fat [5], causing cell damage and organic dysfunction. Furthermore, simultaneous exposure to chronically elevated glucose and FFA concentrations has a synergistic effect that causes glucolipotoxicity, leading to the multiple defects observed in MS [6].

Current therapeutic options for MS are limited to treating hypertension, hyperglycaemia, hypertriglyceridemia, and IR individually [3]. Considering that increased fatty acid release is a key point in MS alterations [7], decreasing triglyceride absorption (one of the main origins of FFA) becomes particularly important.

*Ludwigia octovalvis* (Jacq.) P.H. Raven (Onagraceae) is a flowering helophyte herb that is widely distributed throughout tropical and sub-tropical areas around the world. Due to its rapid growth and high seed production, this species is even considered a pest, one which threatens the production of certain crops, such as rice [8]. The boiled extract or juice of the whole plant has traditionally been used by Indian and Mexican healers in the treatment of diabetes mellitus [9,10] as well as other ailments, including oedema, nephritis, skin conditions, and hypertension [11,12,13].

Previous studies have reported the bioactive potential of *L. octovalvis* in vitro and in vivo, such as antiproliferative activity in 3T3-L1 adipocytes [14]. Additionally, glucose uptake in muscle cells and decreased glucose production in liver cells was observed [15]. Treatment with *L. octovalvis* in rats improved glycemic control (due to hypoglycemic and anti-hyperglycemic activity) through the activation of AMPK [15,16]. In addition, this species had one of the strongest α-glucosidase and pancreatic lipase inhibitory activities among twenty three Mexican medicinal plants in a screening study [10]. The biological effect of *L. octovalvis* on α-glucosidase inhibition has been attributed to bioactive compounds such as gallic acid and ethyl gallate, while isoorientin has the strongest inhibitory activity for pancreatic lipase [17]. Regarding its toxicity, there were no adverse effects with oral administration of up to 5000 mg/kg of the methanolic extract in an acute toxicity model or of up to 800 mg/kg in a subacute toxicity model [18].

Although *L. octovalvis* has been shown to have pharmacological activity in some of the characteristic alterations of MS and is traditionally used for the treatment of hypertension and diabetes (two of the main complications of this syndrome which are strongly associated with IR), it has not been studied in a complex model of MS. Therefore, we explored the effects of this plant on MS in a hypercaloric diet-induced murine model.

## 2. Results

### 2.1. Phytochemistry

**Figure 1:**
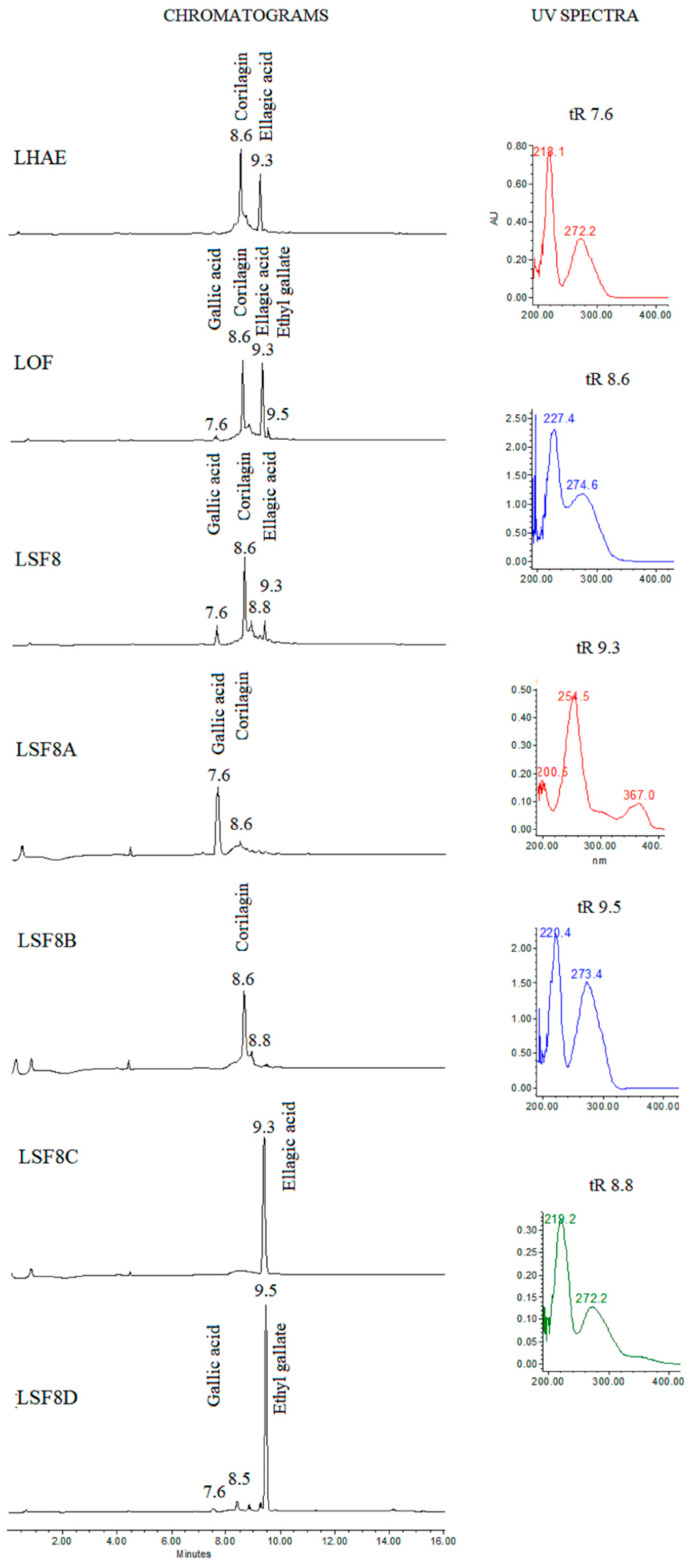
Chromatograms of LHAE and its most-representative subfractions observed at 250 nm, and UV spectra of the major compounds. tR: Retention time.

Thin-layer chromatography of the hydroalcoholic extract (LHAE) and its organic fraction (LOF) showed four main groups of compounds, which, when revealed with natural products-polyethylene glycol (NP-Peg) reagent, were observed in light blue, orange-yellow, black, and bluish purple (Supplementary Material Figure S1). According to HPLC and UV spectra analysis at 250 nm (Figure 1), the sub-fractions with the highest content of these groups of compounds were LSF2, containing mainly coumaroyl derivatives, LSF3, with gallic acid esters, and LSF8 and LSF9, with a mixture in different proportions of gallic acid esters, tannins and traces of flavonoids.

LSF8 fractionation resulted in four sub-fractions with semi-isolated compounds (LSF8A, LSF8B, LSF8C and LSF8D), as shown in Figure 1. When revealed with NP-Peg (Supplementary Material Figure S2), thin-layer chromatography of LSF8A and LSF8D showed a purple-blue color at 365 nm and a light aqua color at 254 nm. In cases of ellagic acid (LSF8C) this polyphenol produces a spot in a timid white color. Nuclear magnetic resonance (NMR) data allowed us to establish the identity of the compounds present in these fractions as gallic acid, ethyl gallate, corilagin and ellagic acid, respectively, as described in the following paragraphs and in Supplementary Material Figures S3–S10.

LSF8A: Pale-yellow powder ^1^H NMR (DMSO): 6.89 ppm (2 H, s). ^13^C NMR (DMSO): 108.84 ppm (2 CH), 118.87 ppm (2 C), 137.81 ppm (1 C), 145.57 ppm (1 C) and 168.64 ppm (1 C). 

LSF8B: Off-white powder ^1^H NMR (DMSO): 4.20 ppm (1H, dd, J = 7.1 Hz), 3.94 ppm (1H, dd, J = 8.6, 10.9 Hz), 4.23 ppm (1 H, dd, J = 8.8 Hz), 3.87 ppm (1H, d, J = 7.3 Hz), 4.35 ppm (1H, t, J = 8.2 Hz), 4.59 ppm (1H, m), 6.20 ppm (1H, d, J = 7.25 Hz), 6.49 ppm (1H, s), 6.56 ppm (1H, s) and 7.01 ppm (2H, s). ^13^C NMR (DMSO): 62.2 ppm (1 CH), 63.9 ppm (1 CH2), 71.7 ppm (1 CH), 76.4 ppm (1 CH), 77.6 ppm (1 CH), 92.2 ppm (1 CH), 106.1 ppm (1 CH), 106.9 ppm (1 CH), 108.9 ppm (2 CH), 115.5 ppm (1 C), 115.8 ppm (1 C), 118.7 ppm (1 C), 135.4 ppm (1 C), 135.5 ppm (2 C), 138.9 ppm (1 C), 143.9 ppm (1 C), 144.3 ppm (2 C), 144.8 ppm (2 C), 145.6 ppm (2 C), 164.8 ppm (1 C), 166.7 ppm (1 C) and 167.1 ppm (1 C).

LSF8C: Off-white powder ^1^H NMR (DMSO): 7.41 ppm (2 H, s). ^13^C NMR (DMSO): 113.08 ppm (2 C), 136.71 ppm (2 C), 141.84 ppm (2 C), 148.86 ppm (2 C), 109.92 ppm (2 CH), 108.69 ppm (2 C) and 159.82 ppm (2 C).

LSF8D: Pale-yellow powder ^1^H NMR (Methanol): 7.05 ppm (2 CH, s), 4.27 ppm (1 H, q, J = 7.4 Hz) and 1.24 ppm (1H, t, J = 7.1 Hz). ^13^C NMR (DMSO): 119.53 ppm (1 C), 107.76 ppm (2 CH), 144.22 ppm (2 C), 137.44 ppm (1 C), 166.32 ppm (1 C), 59.44 ppm (1 CH2) and 12.4 ppm (1 CH3).

NMR data were compared to literature descriptions of gallic acid [19], corilagin [20], ellagic acid [21] and ethyl gallate [22]. All of the isolated compounds had been previously identified in *L. octovalvis* [17,23], except for corilagin, which was, to our knowledge, isolated here for the first time in the genus *Ludwigia*.

### 2.2. MS Model

**Figure 2:**
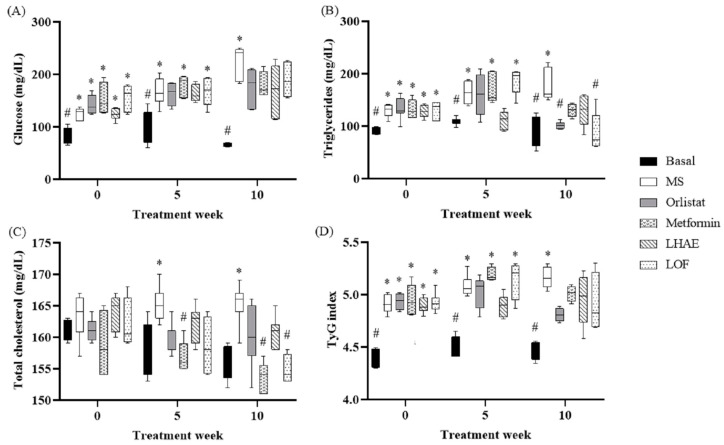
Blood glucose (**A**), triglycerides (**B**), total cholesterol (**C**) and TyG index (**D**) through treatment administration. Basal: Standard diet; MS: Metabolic syndrome (negative control); Orlistat: Commercial drug 40 mg/kg (positive control); Metformin: Commercial drug 100 mg/kg (positive control); LHAE: 25 mg/kg *Ludwigia octovalvis* hydroalcoholic extract; LOF: 25 mg/kg *L. octovalvis* organic fraction. Values are expressed as median and interquartile interval (*n* = 6). Kruskal–Wallis test followed by Dunn’s test for multiple comparisons. * *p* < 0.05 (compared to the basal group), # *p* < 0.05 (compared to the MS group).

**Figure 3:**
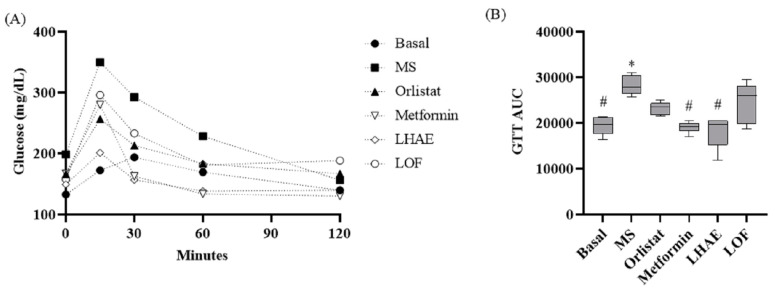
Time course (**A**) and area under the curve (**B**) of the glucose tolerance test after ten weeks of treatment administration. Basal: Standard diet; MS: Metabolic syndrome (negative control); Orlistat: Commercial drug 40 mg/kg (positive control); Metformin: Commercial drug 100 mg/kg (positive control); LHAE: 25 mg/kg *Ludwigia octovalvis* hydroalcoholic extract; LOF: 25 mg/kg *L. octovalvis* organic fraction. Each point in (**A**) represents a mean, while values in (**B**) are expressed as median and interquartile intervals (*n* = 6). Kruskal–Wallis test followed by Dunn’s test for multiple comparisons. * *p* < 0.05 (compared to the basal group), # *p* < 0.05 (compared to the MS group).

**Figure 4:**
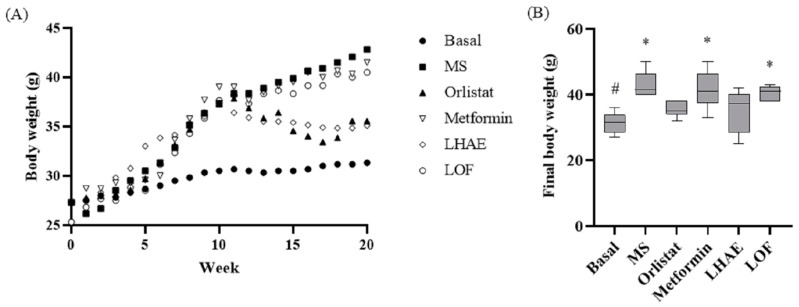
Body weight change throughout metabolic syndrome induction and treatment administration (**A**) and final body weight (**B**). Basal: Standard diet; MS: Metabolic syndrome (negative control); Orlistat: Commercial drug 40 mg/kg (positive control); Metformin: Commercial drug 100 mg/kg (positive control); LHAE: 25 mg/kg *Ludwigia octovalvis* hydroalcoholic extract; LOF: 25 mg/kg *L. octovalvis* organic fraction. Each point in (**A**) represents a mean, while the values in (**B**) are expressed as median and interquartile intervals (*n* = 6). Kruskal–Wallis test followed by Dunn’s test for multiple comparisons. * *p* < 0.05 (compared to the basal group), # *p* < 0.05 (compared to the MS group).

**Figure 5:**
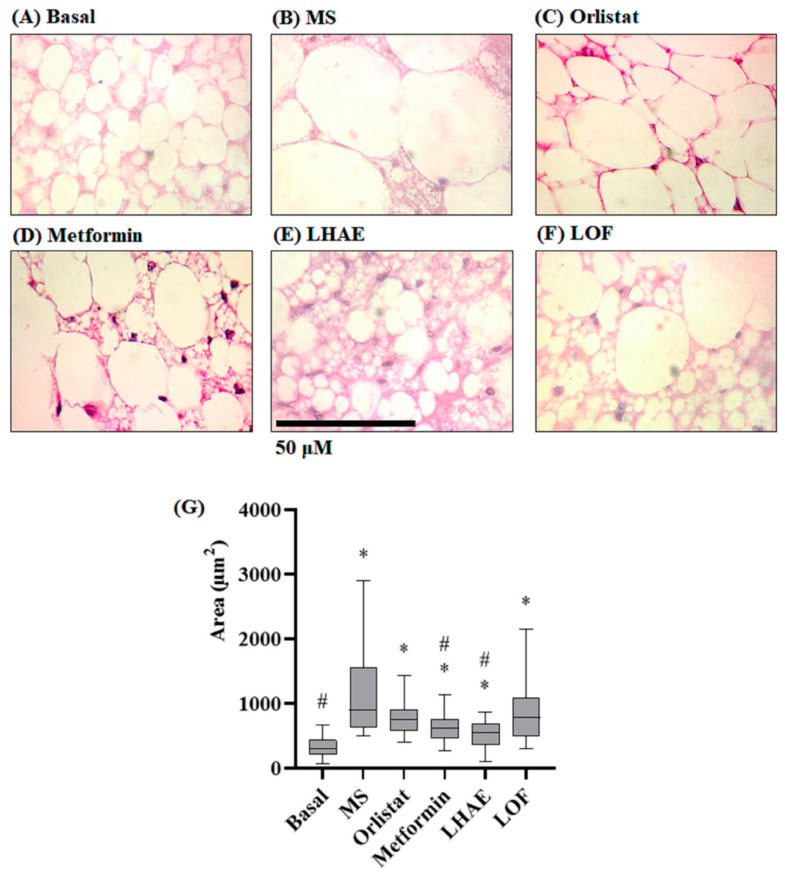
Histology of the perirenal tissue (**A**–**F**) and size of the perirenal adipocytes (**G**) after ten weeks of treatment administration. Perirenal tissue was processed by hematoxylin and eosin staining for histopathological analysis. Microphotographs taken with the 100× objective are representative of each group (*n* = 90 per group). (**A**) Basal: Standard diet; (**B**) MS: Metabolic syndrome (negative control), (**C**) Orlistat: Commercial drug 40 mg/kg (positive control), (**D**) Metformin: Commercial drug 100 mg/kg (positive control); (**E**) LHAE: 25 mg/kg *Ludwigia octovalvis* hydroalcoholic extract; (**F**) LOF: 25 mg/kg *L. octovalvis* organic fraction. The data in the graph (**G**) represent median and interquartile intervals. Kruskal–Wallis test followed by Dunn’s test for multiple comparisons. * *p* < 0.05 compared to the Basal group, # *p* < 0.05 compared to the MS group.

**Figure 6:**
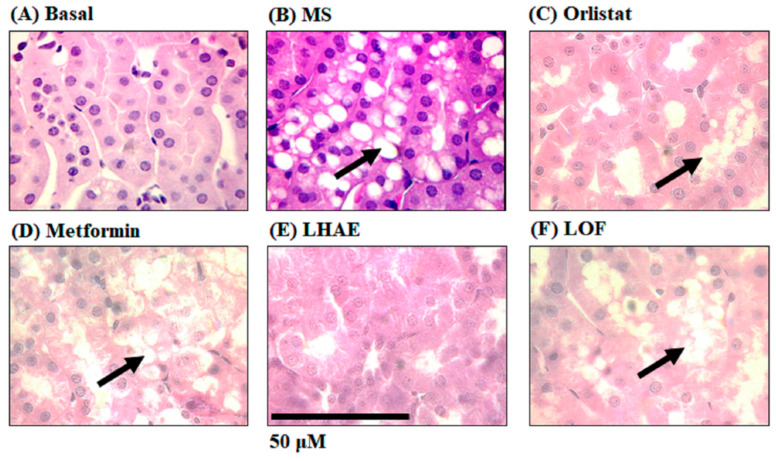
Histology of the kidney to analyse ectopic accumulation of fat in the proximal tubules after ten weeks of treatment administration. The kidneys were processed by hematoxylin and eosin staining for histopathological analysis. Microphotographs taken with the 100× objective are representative of findings in each group (*n* = 6 per group). Arrows indicate increased fat accumulation. (**A**) Basal, (**B**) MS: Metabolic syndrome, (**C**) Orlistat, (**D**) Metformin, (**E**) LHAE: *Ludwigia octovalvis* hydroalcoholic extract, (**F**) LOF: *L. octovalvis* organic fraction.

**Figure 7:**
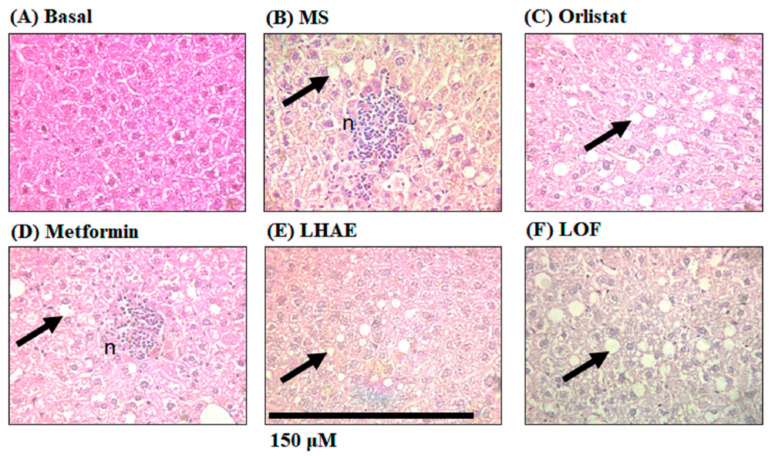
Liver histology to analyse steatosis and liver damage after ten weeks of treatment administration. The liver was processed by hematoxylin and eosin staining for histopathological analysis. Microphotographs taken with the 40× objective are representative of each group (*n* = 6 per group). Arrows indicate increased fat accumulation. n: Lymphocytic micro abscesses with central necrosis. (**A**) Basal, (**B**) MS: Metabolic syndrome, (**C**) Orlistat, (**D**) Metformin, (**E**) LHAE: *Ludwigia octovalvis* hydroalcoholic extract, (**F**) LOF: *L. octovalvis* organic fraction.

According to the data shown in Table 1 and Figure 2, Figure 3, Figure 4, Figure 5, Figure 6 and Figure 7, the hypercaloric diet caused alterations in the MS group in all the parameters evaluated, except for blood cholesterol, proving that the pharmacological model is useful to simulate MS.

#### 2.2.1. IR and Glucose Metabolism

Regarding the basal blood glucose concentration shown in Figure 2, after 10 weeks of treatment, glucose levels tended to decrease in both experimental treatments and positive controls. However, no significant differences were found in comparison with the MS group. The same effect was observed in IR evaluation using the TyG index, which is also shown in Figure 2. 

In the glucose tolerance test (GTT) curve shown in Figure 3, LHAE treatment kept glycemia controlled throughout the whole test to a higher degree than did either of the pharmacological controls; however, this effect was not observed with the administration of the LOF. Area under the curve (AUC) analysis of these graphs showed that the LHAE group value was significantly lower than that observed in the MS group.

#### 2.2.2. Obesity

The change in body weight (BW) over 10 weeks of treatment is presented in Figure 4. After one week with LHAE, there was a decrease in BW similar to that in orlistat administration, and the BW remained similar between these two groups for the remainder of the experiment. Treatment with LOF did not show any effect on BW. Final BWs in the LHAE and orlistat groups were approximately 6 g lower than in the rest of the groups (except the basal group). The effects on BW were not related to changes in the length of the mice (nose-to-anus distance) or food consumption, both of which did differ significantly among groups (data not shown).

The administration of the hypercaloric diet in the MS group also caused a statistically significant increase compared to the basal group in the relative weight of each of the adipose tissue pads, as well as in the total adipose tissue (TAT) and body adiposity index (BAI), as shown in Table 1.

In the LHAE group, body weight loss was caused by decreases in the mesenteric adipose tissue (MAT) to values that were statistically indistinguishable from those of the basal and orlistat groups. The other adipose tissue pads, and therefore the overall BAI, remained statistically similar to that of the MS group, though TAT did decrease significantly, and as sharply as in the orlistat group. LOF did not show important effects in adipose tissue pad weights. 

Histological analysis of the perirenal tissue (Figure 5) showed that the adipocytes of the mice that received the hypercaloric diet (Figure 5B) had statistically significant hypertrophy (increase in individual size) compared to the basal group (Figure 5A), while the groups that received the hypercaloric diet together with some treatment significantly decreased this hypertrophy (Figure 5C–F). The group treated with LHAE did not demonstrate the increase in the size of the adipocytes, despite the consumption of the hypercaloric diet, such that values were statistically lower than in the MS group (see Figure 5G).

The histological analysis of the kidney (Figure 6) showed ectopic accumulation of fat (arrows) in the proximal tubules in all of the groups that received a hypercaloric diet (Figure 6B–D,F), except for the LHAE group (Figure 6E), where no fat deposition was observed, which was similar to the basal group (Figure 6A).

On the other hand, histological analysis of the liver (Figure 7), showed that all the hepatocytes of the mice that received the hypercaloric diet contained fat vesicles (Figure 7B–F). However, unlike the rest of the groups, the MS group (Figure 7B) and metformin group (Figure 7D) presented lymphocytic micro-abscesses with central necrosis (n), indicating more severe liver damage compared to the other treatments.

Triglyceride (TG) concentrations in the liver, in conjunction with its values in heart and kidney (see Table 1), showed that both *L. octovalvis* treatments achieved overall improvement in the three organs as compared to the MS group, to a degree that was similar to that in the pharmacological controls. Related results are observed in heart TG concentrations, while there is a slightly better response with LHAE in kidney TG concentrations.

#### 2.2.3. Atherogenic Dyslipidemia

As can be seen in Figure 2, the TG decreased markedly from the fifth week of treatment, and from that point to the end of the LHAE administration, to values equal to those of the basal group.

LOF, on the other hand, did not show an effect at week 5 of treatment, but did decrease the values in the final evaluation to a degree that was statistically indistinguishable from that of the basal group, as did the two control drugs.

Regarding cholesterol levels (Figure 2), due to the composition of the diet, the increase in the MS group was mild and was not statistically significant until week 5. By 10 weeks of treatment, the LOF showed a decrease in total cholesterol levels similar to that of the metformin group.

#### 2.2.4. Hypertension

There was a statistically significant increase in systolic blood pressure (SBP) and diastolic blood pressure (DBP) in the MS group relative to the basal group, by approximately 20 and 12 mmHg, respectively, as shown in Table 2. 

All of the experimental treatments and pharmacological controls counteracted these increases in SBP to a degree that was statistically similar among all the groups. For DBP, there was also a decrease in the experimental treatments relative to the MS group; in this case, the effect was stronger in the LOF than in the LHAE group, reaching values that were similar to the metformin and basal groups. 

#### 2.2.5. Global Damage Parameters

All the treatment groups showed an improvement in FFA levels compared to the MS group (see Table 3), but LHAE had the strongest effect on this parameter, with levels that were statistically similar to those of the basal group.

When analyzing liver enzymes (Table 3), aspartate aminotransferase (AST) differed significantly only between the basal and MS, demonstrating that AST concentration was successfully controlled by LHAE, LOF and orlistat alike. In the case of alanine aminotransferase (ALT), no statistically significant differences were found in any of the groups, although there was a trend towards a decrease in the LHAE, LOF and orlistat groups.

### 2.3. Postprandial Triglyceridemia

To determine whether the effects observed with the administration of LHAE and LOF were due in part to a decrease in postprandial triglyceridemia, and to determine which of its chemical compounds had the strongest effect on this factor, the four sub-fractions of *L. octovalvis* were evaluated.

**Figure 8:**
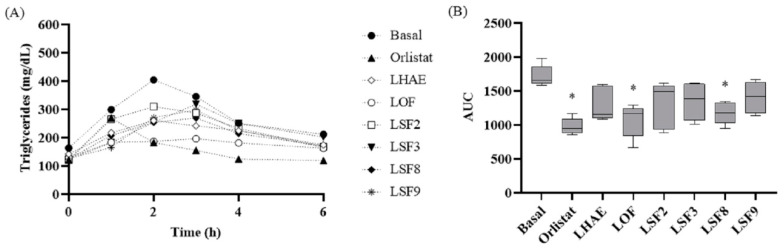
Time course (**A**) and area under the curve (**B**) of the postprandial triglyceridemia test. Basal: No treatment (negative control); Orlistat: Commercial drug 40 mg/kg (positive control); LHAE: 25 mg/kg *Ludwigia octovalvis* hydroalcoholic extract; LOF: 25 mg/kg *L. octovalvis* organic fraction; LSF2-LSF9: 25 mg/kg sub-fractions 2, 3, 8 and 9 of *L. octovalvis* organic fraction. Each point in (**A**) represents a mean, while values in (**B**) are expressed as median and interquartile intervals (*n* = 6). Kruskal–Wallis test followed by Dunn’s test for multiple comparisons. * *p* < 0.05 (compared to the basal group).

The effects of these sub-fractions on postprandial TG concentration is shown in Figure 8, which shows that all treatments tended towards a decrease in TG. However, according to the AUC calculation, the only treatments with a statistically significant decrease were LOF and LSF8. 

## 3. Discussion

The *Ludwigia octovalvis* treatments had significant anti-hyperglycemic, anti-obesogenic and insulin-sensitizing effects. Except for the anti-hyperglycemic effect [15,24], these pharmacological activities had not been previously described in vivo.

LHAE improved GTT, body weight and MAT weight as much or more than did metformin; however, LOF did not show this effect. The fact that the ratios of corilagin with respect to gallic and ellagic acid are the main difference between LOF and LHAE suggests that the main compound responsible for these effects may be corilagin. 

It has been reported that corilagin has anti-hyperglycemic effects in vivo in streptozotocin-induced diabetic rats [25] and in rats with dehydroepiandrosterone-induced polycystic ovary syndrome [26]. Corilagin has also been shown in vitro to inhibit the activity of mammalian intestinal α-glucosidases, as well as enhance PPARγ signaling [20]. Both pharmacological strategies are currently used clinically to treat type 2 diabetes mellitus using the drugs acarbose and pioglitazone, reducing the absorption of carbohydrates from the diet and improving insulin resistance, respectively. Corilagin has been shown in vitro to increase preadipocytes differentiation and decrease lipolysis [27]; this activity could help to reduce lipotoxicity and therefore insulin resistance in MS. The anti-obesity effect of corilagin has not been widely described, except for the decrease in body weight gain induced by polycystic ovary syndrome in rats [26].

On the other hand, LOF had better effects than did LHAE on the control of blood pressure, cholesterol and TG, and therefore with respect to the TyG index, suggesting that the compounds most responsible for improving fat metabolism are ellagic acid and, to a lesser extent, gallic acid. In some mouse models involving insulin resistance, ellagic acid was found to improve some parameters, such as serum glucose/insulin balance, insulin signaling, autophosphorylation, adiponectin receptors, glucose transporters and apoptotic markers in glucose-sensitive tissues, resistin, lipid profile and hepatic steatosis [28,29]. Meanwhile, gallic acid has been shown to prevent streptozotocin-induced hyperlipidemia and hypertension [30].

LHAE and LOF improved the TG concentration in organs almost equally. TG concentrations in the heart and kidneys are directly involved in the pathogenesis of cardiovascular disease, while TG in the liver is involved with cirrhosis development [31,32]. Therefore, these treatments may have potential in the prevention of these complications of MS.

Regarding the parameters of global damage, both LHAE and LOF decreased FFA concentration (33 and 19%) and reduced the liver enzyme AST (67 and 70%), compared to the MS group. This improvement is particularly important because elevated FFA can impair vascular health, acting through multiple aspects of MS, and as discussed above, through the effects of steatosis on multiple tissues. Furthermore, it should be specifically noted that it directly impacts the vascular endothelium, leading to endothelial dysfunction [33]. 

We decided to measure liver enzymes in this model after observing the effects of the treatments on the amount of TG in the liver, which could explain some previously reported hepatoprotective effects of *L. octovalvis* [34]. As there were no significant differences in ALT concentration among the groups, we infer that the hypercaloric diet did not cause severe damage to the liver, although it did produce the onset of necrosis, possibly caused by lipotoxicity [35]. The elevated AST levels after MS induction indicated there was generalized damage in several organs which was diminished by both experimental treatments.

The effects of *L. octovalvis* found in this MS model are due, at least in part, to an improvement in postprandial trigliceridemia, an improvement caused mainly by the corilagin content present in a higher proportion in the LSF8 fraction. Postprandial hypertriglyceridemia (as well as postprandial hyperglycaemia) have the same or even more profound metabolic consequences as fasting triglyceride (and glucose) levels, and they are associated with an increased risk of future cardiovascular events, peripheral vascular disease and cerebrovascular events, as well as the development of diabetes mellitus and obesity [36]. Postprandial hypertriglyceridemia is also a significant cause of lipotoxicity.

Our results have demonstrated that both LHAE and LOF treatments contain bioactive compounds, including coumaroyl derivatives, gallic acid esters, tannins and flavonoids. These compounds have been associated with the observed biological effects, which can act individually or synergistically through several mechanisms. Previous studies have indicated that certain compounds, such as gallic acid and ethyl gallate, could decrease lipid absorption from the diet by inhibiting pancreatic lipase [17], which influences lipid metabolism, and that this could explain the effects found in healthy mice [37], as well as the results in this study.

Furthermore, other studies have reported that *L*. *octovalvis* ethanol extracts induce the activation of the AMPK pathway [14,15]. This pathway plays a crucial role in lipid metabolism, promoting hepatic fatty acid oxidation and inhibiting cholesterol synthesis, lipogenesis, and triglyceride synthesis [38]. 

According to other authors, the administration of corilagin in mice showed an ability to modulate the genetic expression profiles of liver genes induced by a high-fat diet [39].

Moreover, other compounds identified in *L*. *octovalvis* extracts, such as gallic and ellagic acid, have shown inhibitory effects on lipid and triglyceride accumulation. These compounds also downregulated the expression of several adipogenic and lipogenic markers, including PPARy, C/EBPα, C/EBPβ, SREBP-1c, FASN and DGAT1 [40]. 

Further studies are required to explore additional pathways of action of *L. octovalvis* extracts.

## 4. Materials and Methods

### 4.1. Plant Material and Phytochemical Procedures 

The plant material was collected in the state of Morelos and authenticated by MSc Gabriel Flores Franco, curator of the CIByC-UAEM Herbarium (voucher number 34667). The plant’s name was checked and updated with the online website http://www.theplantlist.org, accessed on 15 April 2023 [41].

Using 745 g of dry leaves, we carried out a hydroalcoholic maceration with 60% ethanol to obtain 230 g of LHAE. Afterward a liquid–liquid separation of LHAE was performed under procedures identical to those previously reported [42]. This resulted in 35 g of an organic fraction, referred to as LOF. This fraction was subjected to chemical fractionation by open column chromatography, using silica gel 60 (109385, Merck KGaA, Darmstadt, Germany) as the stationary phase and a hexane/ethyl acetate gradient system as the mobile phase.

To select the four representative sub-fractions of LOF (LSF2, LSF3, LSF8 and LSF9), we performed thin layer chromatography using aluminium sheets with Silica gel 60 RP-18 F254s (105560, Merck KGaA, Darmstadt, Germany), developed with NP-Peg, which consists of a 1% diphenylboryloxyethylamine methanolic solution followed by a 5% polyethylene glycol solution) or ceric sulfate (1% dissolved in sulfuric acid).

After chemical fractionation, we conducted an HPLC analysis. These analyses were performed on Waters brand equipment fitted with a Waters 2996 UV (900) photodiode array detector at 280 nm using Empower 3 software and a SUPELCOSIL packed column (Supelco, St. Louis, MO, USA. LC-F^®^, 25 cm × 4.6 mm; 5 µm) using a trifluoroacetic acid/acetonitrile system as the mobile phase.

LSF8 was subjected to chemical fractionation using the same methods mentioned above until four representative sub-fractions were obtained, namely, LSF8A, LSF8B, LSF8C and LSF8D, together with semi-isolated compounds. The structures of the compounds were elucidated using NMR analysis on an Agilent DD2 (Agilent Technologies, USA) 600 MHz device.

### 4.2. Experimental Animals and MS Induction

Male C57BL6 mice were used for all experiments. They were kept at 25 ± 3 °C under a 12 h:12 h light:dark photoperiod. Food (2028S, 18% Protein Rodent Diet, Harland Tekland) and water were available ad libitum. Animal handling and care was in accordance with internationally accepted procedures (Official Mexican Standard NOM-062-ZOO-1999, technical specifications for the production, care, and use of laboratory animals). This project was approved by the Comité Nacional de Investigación en Salud del Instituto Mexicano del Seguro Social (R-2019-785-088).

The mice were randomly divided into study groups of ten individuals each (*n* = 10), upon reaching a BW of 25 to 30 g (between the fifth and seventh week). All groups except for the basal group were then started on a hypercaloric diet ad libitum for 10 weeks. The hypercaloric diet treatment was carried out as reported by [43]. In the animals within the experimental groups that achieved an increase of at least 15% in BW with respect to the basal group, the administration of the assigned diet was continued for 10 more weeks, together with the daily oral treatment that corresponded to each group, as described in Table 4.

Mice used in the postprandial triglyceridemia test were randomly divided into study groups of five individuals each (*n* = 5), all of which were fed the standard diet ad libitum.

The animals were sacrificed according to methods described in NOM-062-ZOO-1999. For the MS model, at the end of the 20 weeks of treatment, and after evaluating all the necessary parameters, the mice were placed under deep anesthesia by administration of pentobarbital 65 mg/kg intraperitoneally, and complete exsanguination was conducted through the infraorbital sinus of each mouse to obtain the final blood sample. In the case of the animals used in the postprandial triglyceridemia test, an overdose of anesthesia was used.

### 4.3. Evaluated Parameters in the MS Model

Glucose, total cholesterol and triglyceride TG concentrations were evaluated in a blood sample taken after 4 h of fasting, once at the end of MS induction and a second time during week 15 of the experiment, using the Accu-Chek Performa and Accutrend Plus measuring devices (Roche, Basel, Switzerland).

At the end of the experiment, serum was obtained by centrifuging the blood from the infraorbital sinus. Glucose and TG concentrations in serum were quantified using BioSystems clinical kits 11803 and 11828. FFA concentrations in serum were determined using the method reported by Falholt et al. [44].

The TyG index was used to indirectly detect IR [45] based on the concentrations of glucose and TG, using the formula:

TyG = ln (Triglycerides * Glucose)/2(1)

The glucose tolerance test was conducted at the end of the experiment by measuring blood glucose levels after 4 h of fasting and then 15, 30, 60, 120 and 180 min after the oral administration of the corresponding treatment and a glucose solution at 2 g/kg. With the obtained data, a graph was constructed and the AUC was calculated.

Blood pressure was measured at the end of the experiment by a non-invasive method with an LE 5002 Storage pressure meter (Letica). The animals of each treatment group were sedated by administering a low dose of pentobarbital (10 mg/kg) intraperitoneally and heated at 35 ± 2 °C for 5 min; then, an insufflator ring attached to a transducer (Biopac System MP150) was placed at the base of the tail. An average of 10 consecutive stable measurements were recorded for each mouse (with a difference of 1 min) and then averaged to obtain the systolic and diastolic pressure of each individual.

ALT and AST concentrations were determined at the end of the experiment from the serum obtained by centrifugation of blood from the infraorbital sinus using BioSystems AST/GOT IFCC (11830) and ALT/GTP IFCC (11832) clinical kits.

After sacrifice, each mouse was perfused with ice-cold PBS (NaCl 140 mM, KCl 2 mM, and K_2_HPO_4_ 1.15 mM) in order to subsequently remove and weigh the kidneys, liver, and heart, as well as the mesenteric, epididymal, subcutaneous, and perirenal adipose tissue pads. The weights of the adipose tissue pads of each mouse were summed to calculate the TATs, which were then used to calculate the BAI according to the formula:

BAI (%) = (TAT weight/Body weight) * 100.(2)

The heart, as well as half of the liver and kidney samples, were frozen at −80 °C for TG quantification, while the second half of the liver and kidney samples were stored in 10% formalin for histological preparations.

For the histopathological analysis, after one week in formalin, the liver and kidneys were placed in cassettes, dehydrated, and embedded in paraffin. The obtained blocks were cut into 6 μm sections using a microtome. The sections were recovered on a glass slide, deparaffinized and hydrated, and finally stained with hematoxylin-eosin. The microphotographs were acquired with a Nikon ECLIPSE 80i microscope and analyzed with the Metamorph software, v. 6.1.

TG was quantified in the frozen heart, liver and kidneys using a previously reported extraction method [46]. Briefly, each organ was ground and washed with 0.2 volumes of saline solution (0.9% NaCl), then centrifuged at 4000× *g* for 10 min. The lipid phase was then recovered, and the TG content was determined using an enzymatic kit (Randox Laboratories Ltd.).

### 4.4. Postprandial Triglyceridemia

This test was carried out in seven-week-old mice that were divided into groups of 5 individuals (*n* = 5) following a methodology previously reported [47]. Briefly, after 6 h of fasting, the basal TG levels were measured with the Accutrend Plus measuring device (Roche), and then 100 μL of vehicle (5% Tween), orlistat (40 mg/kg), or the corresponding experimental treatment (sub-fractions of *L. octovalvis*) at a dose of 25 mg/kg was administered orally. Next, 400 µL of olive oil was administered orally and TG levels were measured after 1, 2, 3, 4 and 6 h. A response curve was constructed with the data obtained and the area under the curve was calculated.

### 4.5. Data Processing and Statistical Aspects

The results in graphs are expressed as median and interquartile intervals, while the results in tables are expressed as the mean ± SD (standard deviation).

The comparison among groups was conducted using Kruskal–Wallis test followed by Dunn’s test. Differences were considered significant when *p* < 0.05.

GraphPad Prism 9.5.1 software was used for AUC calculation, graphing and statistical analysis.

## 5. Conclusions

The hypercaloric diet caused a statistically significant increase in basal glycemia, glucose tolerance, IR, BW, adipose tissue, blood triglycerides and blood pressure, one which decreased with the administration of *L. octovalvis*-based treatments. Although the ethyl acetate fraction was active, the hydroalcoholic extract showed a better overall result due to its greater control of body weight and free fatty acid concentration. These effects are possibly due to its high content of corilagin and ellagic acid. 

## Figures and Tables

**Table 1:** Values of the parameters related to obesity at the end of the administration of treatments.

Parameter	Basal	MS	Orlistat	Metformin	LHAE	LOF
MAT (mg/g)	2.7 ± 2.2 #	22.8 ± 8.2 *	10.2 ± 3.5	20.0 ± 7.9 *	11.0 ± 5.6	17.1 ± 5.3 *
SAT (mg/g)	14.5 ± 12.1 #	93.1 ± 17.1*	38.5 ± 14.3	71.0 ± 16.4 *	64.4 ± 19.4	67.5 ± 12.5 *
PAT (mg/g)	10.9 ± 7.9 #	28.4 ± 8.2 *	19.9 ± 3.9	23.2 ± 2.4	22.2 ± 5.9	23.3 ± 3.6
EAT (mg/g)	26.2 ± 14.4 #	62.8 ± 9.5 *	42.9 ± 3.8	51.2 ± 7.8	52.7 ± 12.2	51.8 ± 11.4
TAT (mg/g)	54.3 ± 20.5 #	207.1 ± 21.3 *	110.5 ± 23.3	165.4 ± 21.4 *	150.2 ± 40.8	159.7 ± 20.2 *
BAI	2.61 ± 1.42 #	6.26 ± 0.97 *	4.31 ± 0.41	5.12 ± 0.79	5.25 ± 1.21	5.18 ± 1.14
LTG (mg/g)	396.5 ± 26.1 #	864.3 ± 31.7 *	604.6 ± 95.9	575.6 ± 155.0	502.3 ± 183.6	733.9 ± 202.7
HTG (mg/g)	1488 ± 124 #	2820 ± 542 *	1921 ± 201	1795 ± 95	901 ± 434	976 ± 393
KTG (mg/g)	1083 ± 670 #	2841 ± 374 *	2249 ± 909	2195 ± 306	965 ± 384 #	2681 ± 471

Basal: Standard diet; MS: Metabolic syndrome (negative control); Orlistat: Commercial drug 40 mg/kg (positive control); Metformin: Commercial drug 100 mg/kg (positive control); LHAE: 25 mg/kg *Ludwigia octovalvis* hydroalcoholic extract; LOF: 25 mg/kg *L. octovalvis* organic fraction. Values are expressed as mean ± SD (*n* = 6). Kruskal–Wallis test followed by Dunn’s test for multiple comparisons. * *p* < 0.05 (in comparison with basal group), # *p* < 0.05 (in comparison with MS group). MAT: mesenteric adipose tissue; SAT: subcutaneous adipose tissue; PAT: perirenal adipose tissue; EAT: epididymal adipose tissue; TAT: total adipose tissue; BAI: body adiposity index; LTG: liver triglycerides; HTG: heart triglycerides; KTG: kidney triglycerides.

**Table 2:** Values of systolic and diastolic blood pressure at the end of the administration of treatments.

Parameter	Basal	MS	Orlistat	Metformin	LHAE	LOF
SBP (mmHg)	89.9 ± 11.9 #	110.3 ± 16.7 *	87.4 ± 7.1 #	86.8 ± 6.4 #	84.3 ± 11.5 #	87.8 ± 9.2 #
DBP (mmHg)	61.6 ± 7.9 #	73.7 ± 8.5 *	53.0 ± 5.0 #	60.2 ± 11.3 #	65.6 ± 3.3	57.5 ± 9.1 #

Basal: Standard diet; MS: Metabolic syndrome (negative control); Orlistat: Commercial drug 40 mg/kg (positive control); Metformin: Commercial drug 100 mg/kg (positive control); LHAE: 25 mg/kg *Ludwigia octovalvis* hydroalcoholic extract; LOF: 25 mg/kg *L. octovalvis* organic fraction. Values are expressed as mean ± SD (*n* = 6). Kruskal–Wallis test followed by Dunn’s test for multiple comparisons. * *p* < 0.05 (in comparison with basal group), # *p* < 0.05 (in comparison with MS group). SBP: systolic blood pressure; DBP: diastolic blood pressure.

**Table 3:** Values of the parameters related to global damage at the end of the administration of treatments.

Parameter	Basal	MS	Orlistat	Metformin	LHAE	LOF
FFA (μg/mL)	26.6 ± 5.4 #	41.9 ± 5.1 *	32.1 ± 3.8	41.3 ± 6.0 *	28.1 ± 2.8	33.9 ± 6.1
AST (U/L)	91.7 ± 34.2 #	227.0 ± 84.4 *	73.1 ± 37.7 #	207.9 ± 113.6	74.2 ± 23.4	68.3 ± 50.6 #
ALT (U/L)	25.6 ± 19.9	39.2 ± 18.4	15.0 ± 7.1	32.1 ± 12.1	13.0 ± 10.2	10.8 ± 1.2

Basal: Standard diet; MS: Metabolic syndrome (negative control); Orlistat: Commercial drug 40 mg/kg (positive control); Metformin: Commercial drug 100 mg/kg (positive control); LHAE: 25 mg/kg *Ludwigia octovalvis* hydroalcoholic extract; LOF: 25 mg/kg *L. octovalvis* organic fraction. Values are expressed as mean ± SD (*n* = 6). Kruskal–Wallis test followed by Dunn’s test for multiple comparisons. * *p* < 0.05 (in comparison with basal group), # *p* < 0.05 (in comparison with MS group). FFA: free fatty acids; AST: aspartate aminotransferase; ALT: alanine aminotransferase.

**Table 4:** Groups in the murine metabolic syndrome model.

Group	Diet	Treatment in 10 to 20 Weeks
Basal	Standard	Vehicle (5% Tween 20)
MS	Hypercaloric	Vehicle (5% Tween 20)
Orlistat	Hypercaloric	Orlistat 40 mg/kg
Metformin	Hypercaloric	Metformin 100 mg/kg
LHAE	Hypercaloric	*L. octovalvis* hydroalcoholic extract 25 mg/kg
LOF	Hypercaloric	*L. octovalvis* organic fraction 25 mg/kg

## Data Availability

Not applicable.

## Supplementary Materials

The following supporting information can be downloaded at: https://www.mdpi.com/article/10.3390/plants12132578/s1, Figure S1: Thin-layer chromatography, without chemical treatment and with Natural Products-Polyethylene Glycol (NP-Peg) reagent, of the hydroalcoholic extract of *Ludwigia octovalvis* LHAE (A), its organic fraction LOF (B) and the most representative subfractions of its chemical content: LSF2 (C), LSF3 (D), LSF8 (E) and LSF9 (F); Figure S2: Thin-layer chromatography, without chemical treatment and with Natural Products-Polyethylene Glycol (NP-Peg) reagent, of the hydroalcoholic extract of *Ludwigia octovalvis* LHAE (A), its organic fraction LOF (B) and the subfractions LSF8A (C), LSF8B (D), LSF8C (E) and LSF8D (F), obtained from *Ludwigia octovalvis* organic fraction; Figure S3: ^1^H NMR spectra of the subfraction obtained from *Ludwigia octovalvis* LSF8A and structure of the compound elucidated therein, gallic acid; Figure S4: ^13^C NMR spectra of the subfraction obtained from *Ludwigia octovalvis* LSF8A and structure of the compound elucidated therein, gallic acid; Figure S5: ^1^H NMR spectra of the subfraction obtained from *Ludwigia octovalvis* LSF8B and structure of the compound elucidated therein, corilagin; Figure S6: ^13^C NMR spectra of the subfraction obtained from *Ludwigia octovalvis* LSF8B and structure of the compound elucidated therein, corilagin; Figure S7: ^1^H NMR spectra of the subfraction obtained from *Ludwigia octovalvis* LSF8C and structure of the compound elucidated therein, ellagic acid; Figure S8: ^13^C NMR spectra of the subfraction obtained from *Ludwigia octovalvis* LSF8C and structure of the compound elucidated therein, ellagic acid; Figure S9: ^1^H NMR spectra of the subfraction obtained from *Ludwigia octovalvis* LSF8D and structure of the compound elucidated therein, ethyl gallate; Figure S10: ^13^C NMR spectra of the subfraction obtained from *Ludwigia octovalvis* LSF8D and structure of the compound elucidated therein, ethyl gallate
