# Supplementary material for: Chemical Characterization, Antilipidemic Effect and Anti-Obesity Activity of Ludwigia octovalvis in a Murine Model of Metabolic Syndrome
Source: Plants (Basel). 2023 Jul 7;12(13):2578. doi: 10.3390/plants12132578 (PMC10346568; doi:10.3390/plants12132578)
Supplement: Supplementary file 1 [file plants-12-02578-s001.zip › plants-2440281-supplementary.pdf]

Chemical Characterization, Antilipidemic Effect and Antiobesity Activity of *Ludwigia octovalvis* in a Murine Model of Metabolic Syndrome

Supplementary material

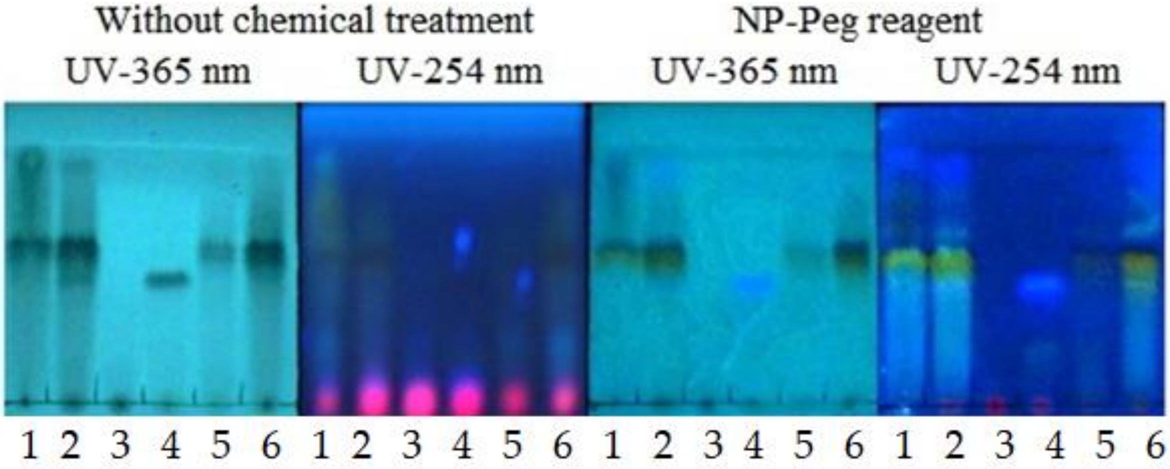

Figure S1. Thin-layer chromatography, without chemical treatment and with Natural Products-Polyethylene Glycol (NP-Peg) reagent, of the hydroalcoholic extract of *Ludwigia octovalvis* LHAE (1), its organic fraction LOF (2) and the most representative subfractions of its chemical content: LSF2 (3), LSF3 (4), LSF8 (5) and LSF9 (6). Mobile phase: water/acetonitrile 70/30.

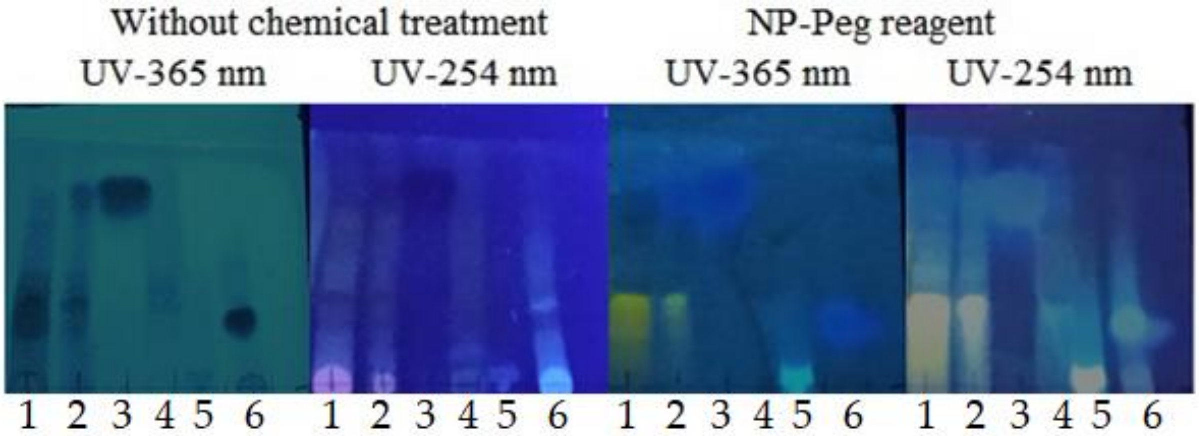

Figure S2. Thin-layer chromatography, without chemical treatment and with Natural Products-Polyethylene Glycol (NP-Peg) reagent, of the hydroalcoholic extract of *Ludwigia octovalvis* LHAE (1), its organic fraction LOF (2) and the subfractions LSF8A (3), LSF8B (4), LSF8C (5) and LSF8D (6), obtained from *Ludwigia octovalvis* organic fraction. Mobile phase: water/acetonitrile 60/40.

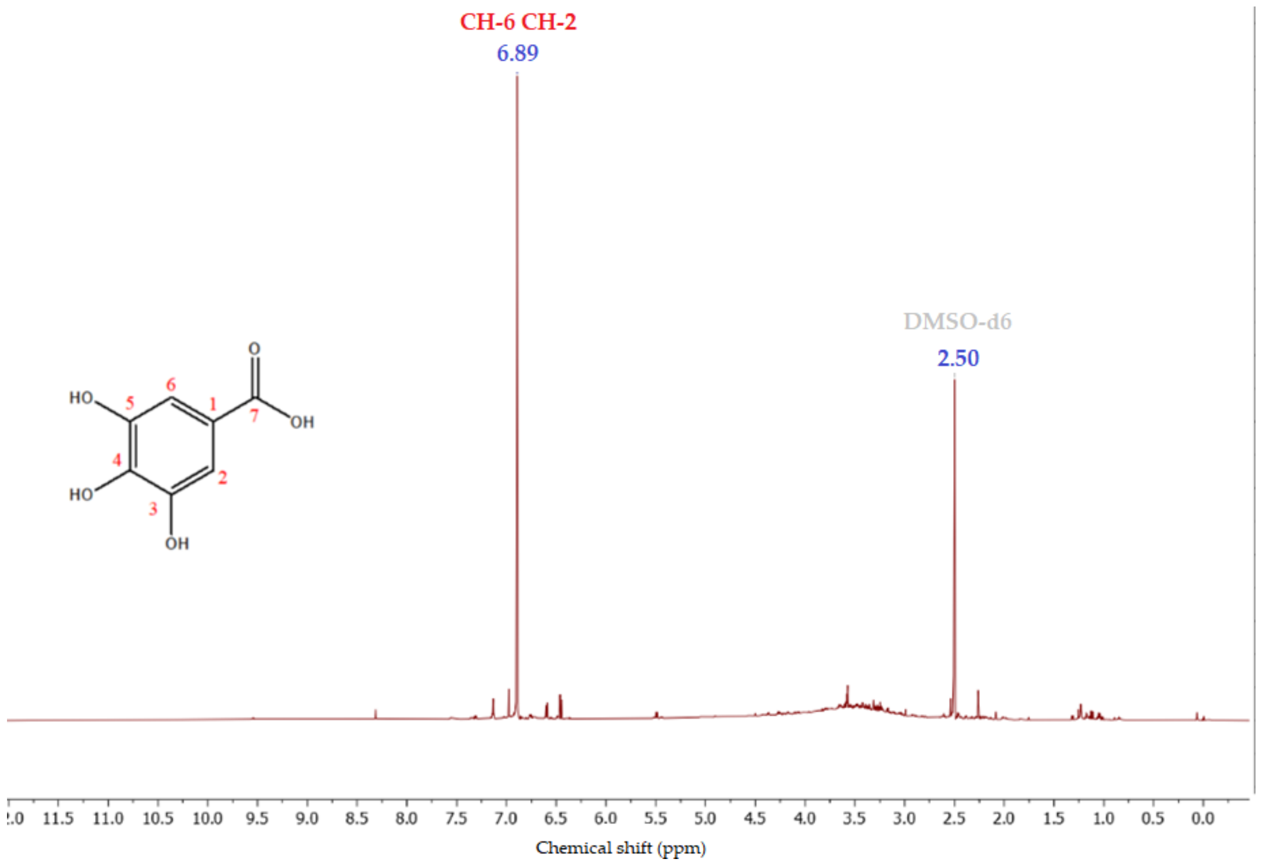

Figure S3. ^{1}H NMR spectra of the subfraction obtained from *Ludwigia octovalvis* LSF8A and structure of the compound elucidated therein, gallic acid.

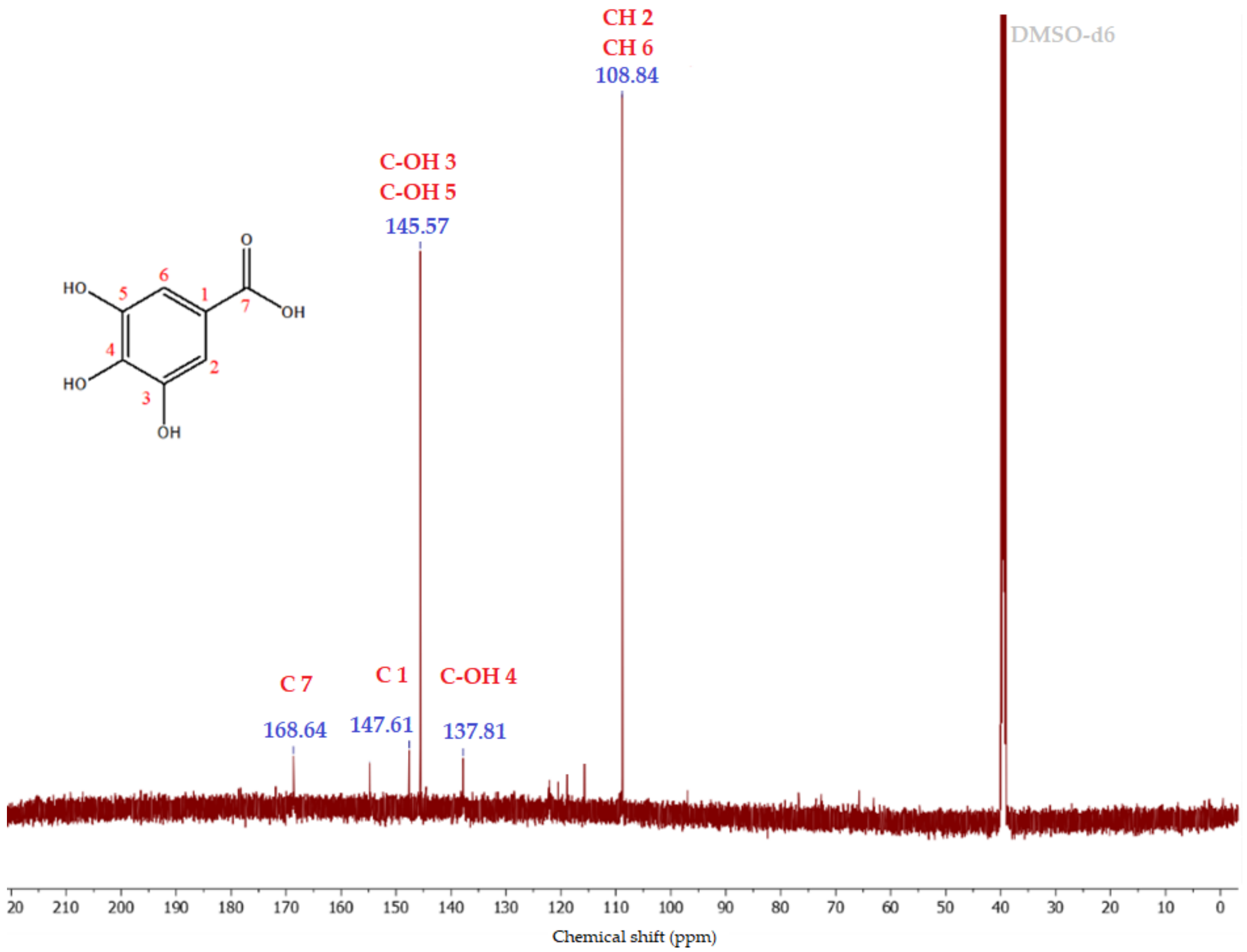

Figure S4. ^{13}C NMR spectra of the subfraction obtained from *Ludwigia octovalvis* LSF8A and structure of the compound elucidated therein, gallic acid.

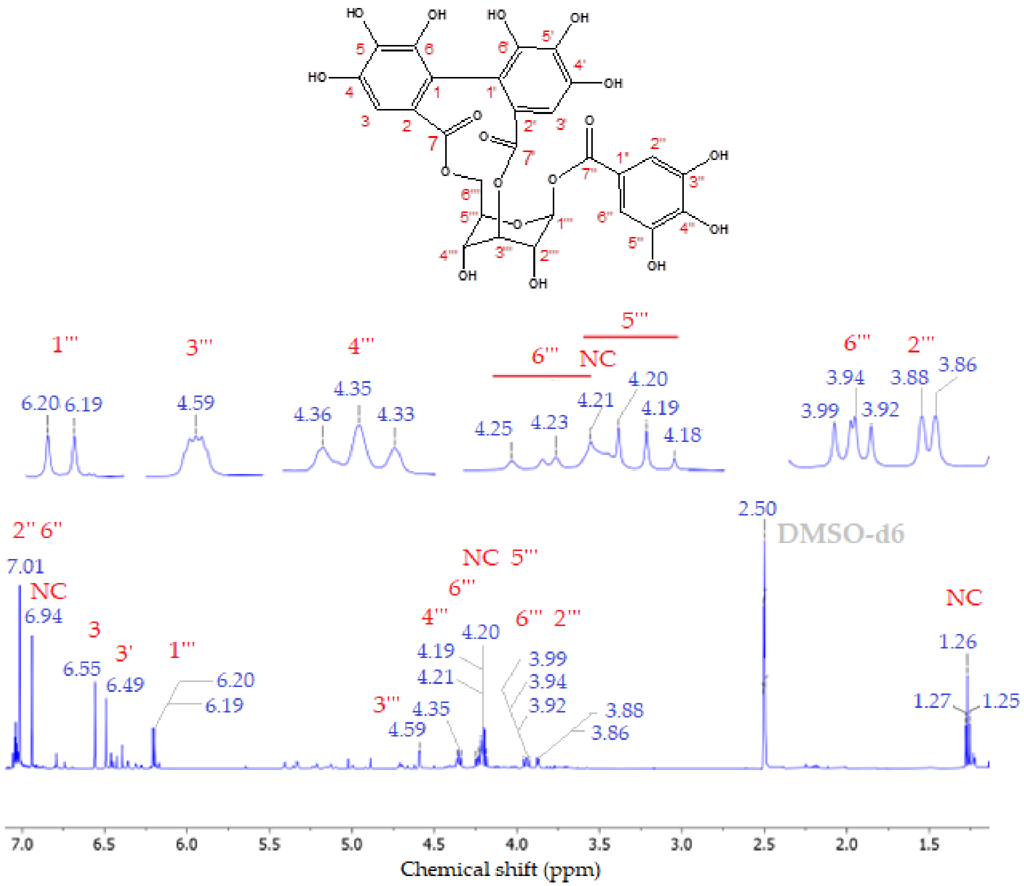

Figure S5. ^{1}H NMR spectra of the subfraction obtained from *Ludwigia octovalvis* LSF8B and structure of the compound elucidated therein, corilagin.

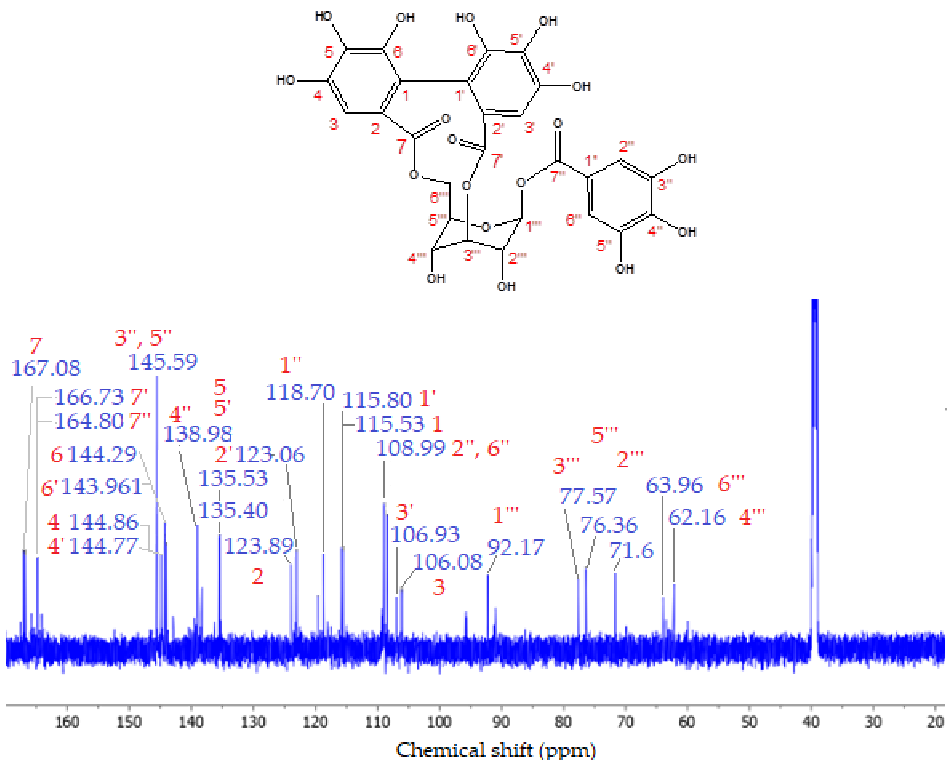

Figure S6. ^{13}C NMR spectra of the subfraction obtained from *Ludwigia octovalvis* LSF8B and structure of the compound elucidated therein, corilagin.

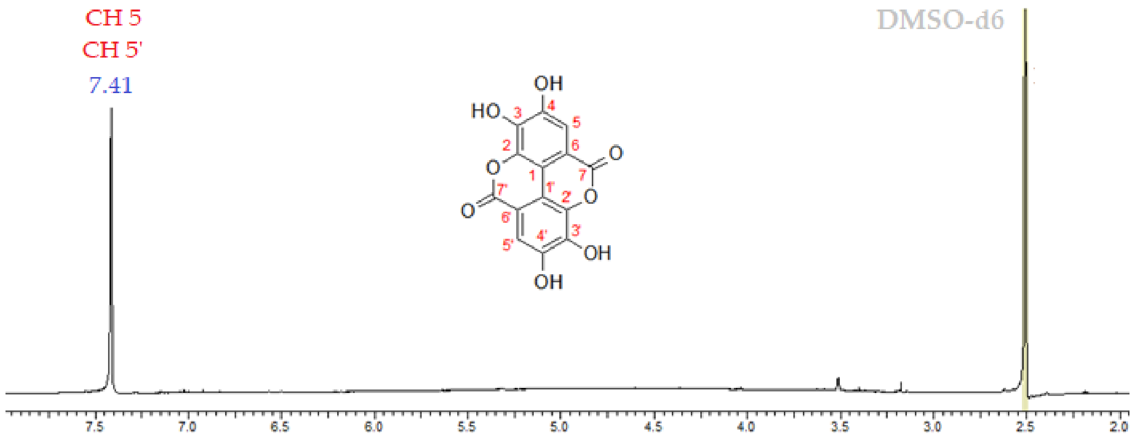

Figure S7. ^{1}H NMR spectra of the subfraction obtained from *Ludwigia octovalvis* LSF8C and structure of the compound elucidated therein, ellagic acid.

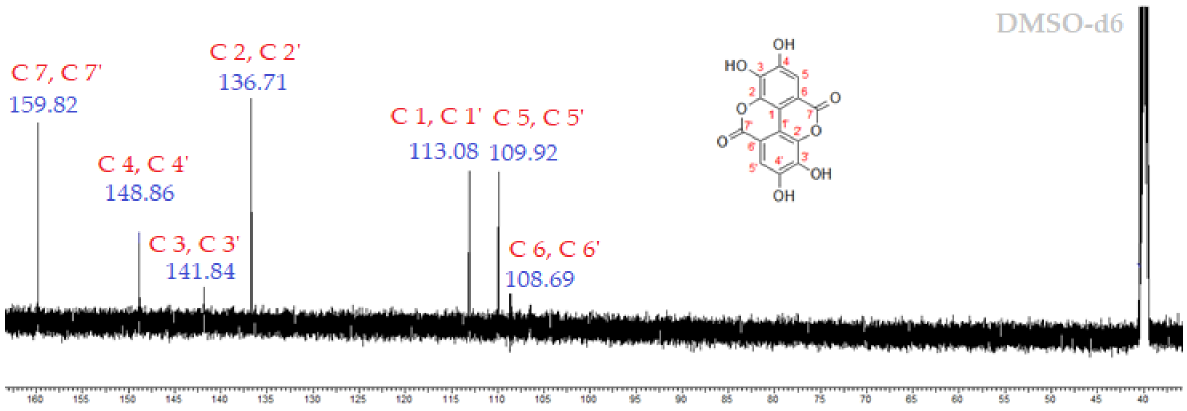

Figure S8. ^{13}C NMR spectra of the subfraction obtained from *Ludwigia octovalvis* LSF8C and structure of the compound elucidated therein, ellagic acid.

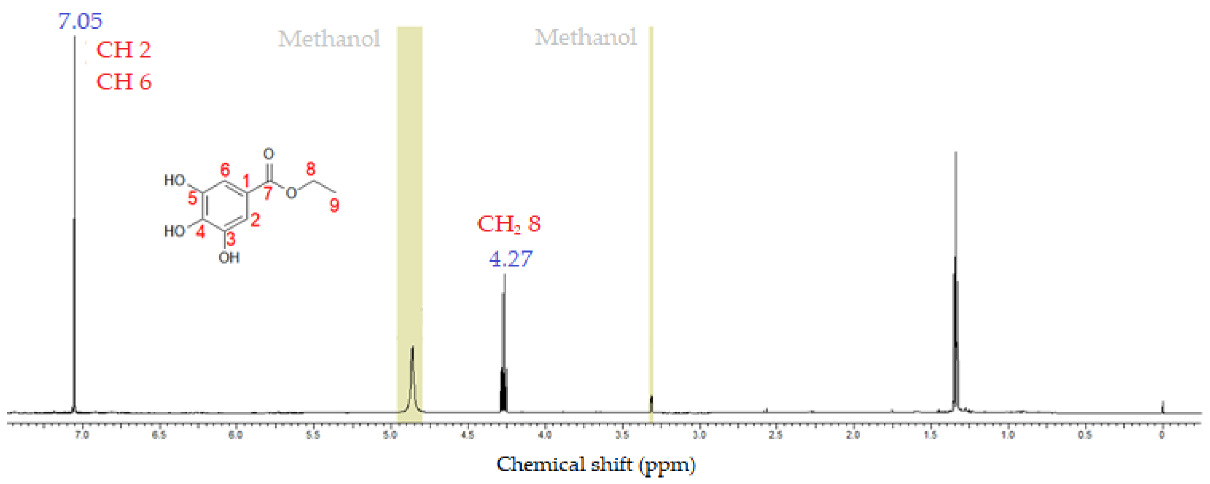

Figure S9. ^{1}H NMR spectra of the subfraction obtained from *Ludwigia octovalvis* LSF8D and structure of the compound elucidated therein, ethyl gallate.

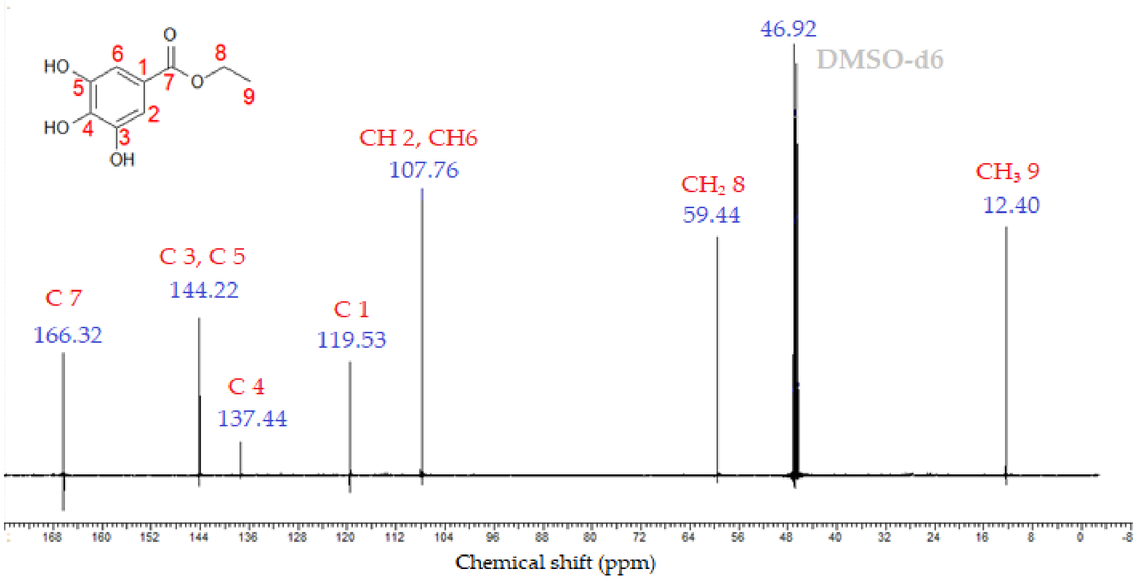

Figure S10. ^{13}C NMR spectra of the subfraction obtained from *Ludwigia octovalvis* LSF8D and structure of the compound elucidated therein, ethyl gallate.
